# A potential immunotherapeutic and prognostic biomarker for multiple tumors including glioma: SHOX2

**DOI:** 10.1186/s41065-023-00279-8

**Published:** 2023-05-11

**Authors:** Xiaocong Wu, Hui Chen, Chao You, Zongjun Peng

**Affiliations:** 1 Department of Neurosurgery, Sichuan Friendship Hospital, 96 Shangshahepu Street, Jinjiang District, Chengdu, Sichuan 610066 China; 2 Department of Neurosurgery, West China Hospital, Sichuan University, 37 Guoxue Lane, Wuhou District, Chengdu, Sichuan 610041 China

**Keywords:** SHOX2, Pan-cancer, Prognosis, Immune, Glioma

## Abstract

**Background:**

Short stature homeobox 2 (SHOX2) is significant gene in the development and progression of multiple types of tumors. Nonetheless, the biological role of SHOX2 within pan-cancer datasets has not been investigated. Thus, comprehensive bioinformatics analyses of pan-cancer datasets were conducted to explore how SHOX2 regulates tumorigenesis.

**Methods:**

A variety of tumor datasets and online analytical tools, including SangerBox, TIMER2, LinkedOmic, GEPIA2 and cBioPortal, were applied to explore SHOX2 expression in various tumors. To ascertain the connections between SHOX2 expression and genetic alterations, SHOX2-related genes and tumor immunity, the pan-cancer datasets were examined. In vitro assays were applied to verify the biological functions of SHOX2 in glioma cells via CCK-8, wound healing, Transwell and colony formation assays.

**Results:**

Analyses found that SHOX2 was overexpressed in multiple cancer types. SHOX2 expression level was significantly correlated with isocitrate dehydrogenase (IDH), 1p/19q, O^6^-methylguanine DNA methyltransferase (MGMT) status and new types of glioma patients. High mRNA expression levels of SHOX2 were associated with a poor prognosis in multiple tumor patients. KEGG enrichment analysis showed that SHOX2-related genes were associated with cell cycle and DNA damage repair. Genetic alterations of SHOX2 were identified in multiple types of cancers, including duplications and deep mutations. Immune analysis showed that SHOX2 was closely correlated with the tumor mutation burden (TMB), microsatellite instability (MSI), neoantigen and neoantigens and immune checkpoint (ICP) in a variety of tumors and could influence the immunotherapy sensitivity of cancers. CCK-8, wound healing, Transwell and colony formation experiments showed that SHOX2 knockdown inhibited glioma cell proliferation, migration, invasion and colony formation abilities.

**Conclusion:**

SHOX2 was overexpressed in multiple cancer types in TCGA cohort. SHOX2 knockdown inhibited glioma cell proliferation, migration and colony formation ability. Our study showed that SHOX2 may be an immunotherapeutic and promising prognostic biomarker in certain types of tumors.

**Supplementary Information:**

The online version contains supplementary material available at 10.1186/s41065-023-00279-8.

## Introduction

Human short stature homeobox gene 2 (SHOX2), also known as SHOT, OG12X or OG12, is homologous to the short stature homeobox gene SHOX [1]. At the amino acid level, SHOX2 shares 99% sequence identity with the murine counterpart, which exhibits similar expression patterns during embryonic development [2].

Several present researches reported that SHOX2 is associated with the tumorigenesis and progression of a variety of cancers. For example, the DNA locus’s hypermethylation of SHOX2 may serve as a biomarker for lung cancer [3–5]. As evidenced by the observation that the expression level of SHOX2 was closely related to cancer recurrence in hepatocellular carcinoma (HCC), SHOX2 might play a significant role in tumorigenesis[6]. For advanced lung cancer, due to the significant copy number variation in SHOX2, increased extracellular methylation SHOX2 DNA could be exploited for diagnosis and prognosis [6]. In addition, in patients with *Helicobacter pylori*-infected gastric cancer, SHOX2 was among the most elevated genes after STAT3 activation, which demonstrated that SHOX2 was involved in the beginning and development of gastric carcinogenesis [7]. Teng et al. found that breast cancer metastasis could be promoted by SHOX2 cooperated with STAT3 through transcriptional regulation of WASF3 [8]. As these findings demonstrate, SHOX2 played an important role in tumorigenesis. However, the comprehensive researches of SHOX2 in various cancers has not been studied at present.

In this research, systematic bioinformatics analyses were performed to verify the prognostic significance and biological functions of SHOX2 in pan-cancers via a variety of datasets. The prognostic, genetic, SHOX2-related gene, and tumor immunity implications of altered SHOX2 expression levels in various malignancies were thoroughly investigated. Through in vitro assays, we specifically examined the relationship between SHOX2 mRNA expression and the development and proliferation of gliomas.

## Materials and methods

### SHOX2 mRNA expression

The Cancer Genome Atlas (TCGA) and Genotype-Tissue Expression (GTEx) can be conducted using SangerBox (http://SangerBox.com/Tool), a valuable online portal [9]. “SHOX2” was input into this web server to determine whether there was a divergence between normal and tumor tissues in SHOX2 mRNA expression via TCGA and GTEx. SHOX2 mRNA expression distributions were visualized in violin plots. The TCGA and GTEx cohorts were applied to explore SHOX2 mRNA expression in 33 tumors (List of abbreviations showed all the abbreviations).

In addition, the mRNA expression levels of SHOX2 were explored in glioma patients with different grades, isocitrate dehydrogenase (IDH) status (mutant or wildtype), 1p/19q status (codeletion or non-codeletion), O^6^-methylguanine DNA methyltransferase (MGMT) status (Methylated or Unmethylated) and new types (oligodendroglioma, astrocytoma and glioblastoma) by the TCGA, CGGA and GSE16011 databases.

### Survival prognosis analysis

In the TCGA database, disease-free survival (DFS) and overall survival (OS) maps of SHOX2 in pan-cancers were obtained via GEPIA2 online website (http://gepia2.cancer-pku.cn/#index) [10]. By applying the expression thresholds of the cutoff-high (50%) and cutoff-low (50%) values, we were able to identify the low-expression and high-expression cohorts of SHOX2. Then, according to the “Survival Analysis” module of GEPIA2, we examined unique survival plots with log-rank *P*-values. In ACC, BLCA, KIRP, KIRC, LIHC, LGG, MESO and STAD, the expression level of SHOX2 had statistically difference from the prognosis of patients in OS. In ACC, KIRP, KIRC and LGG, SHOX2 mRNA expression had statistically difference from the prognosis of patients in DFS. The correlations between SHOX2 mRNA expression and prognosis of glioma patients were analyzed via TCGA, CGGA and GSE16011 datasets. Moreover, the COX_OS analysis of SHOX2 was investigated in multiple cancers by SangerBox online website. Furthermore, the correlations between SHOX2 expression level and prognosis (OS) in gastric cancer (GC), liver cancer, lung cancer and ovarian cancer (OV) were explored via Kaplan-Meier Plotter portal (https://kmplot.com/analysis/). Kaplan-Meier survival plots were generated for gastric, liver cancer, lung and ovarian cases in the “mRNA RNA-seq” and the “mRNA gene chip” modules. The hazard ratio (HR), 95% confidence intervals and log-rank *P*-value were calculated.

### SHOX2-related gene enrichment analysis

Through the “Similar Gene Detection” function, GEPIA2 portal was applied to explore the top eight SHOX2-related genes in TCGA database. GEPIA2 was used to investigate the association between SHOX2 and these top eight genes via the “correlation analysis” module, including CDKN2C, COL6A1, COL6A2, DCHS1, MAPK7, PRRX1, RAB23 and RSRC1. The *P*-value and the correlation coefficient (R) were calculated. Then, a heatmap of the top eight SHOX2-related genes was generated in pan-cancers using the “Gene_Corr” function of TIMER2 (http://timer.cistrome.org/) [11]. The correlations between SHOX2 expression and these 8 related genes were further analyzed in LGG and GBM via TIMER2 website. Then, STRING portal (https://string-db.org/) was used to construct the protein interaction network of top 200 SHOX2-related genes. Moreover, we analyzed the top 50 SHOX2 positively-related genes or SHOX2 negatively-related genes and made heatmaps in glioma via LinkedOmics portal (http://www.linkedomics.org/login.php) [12]. The gene set enrichment analysis (GSEA) function module was used to evaluate the KEGG (Kyoto Encyclopedia of Genes and Genomes) pathways. The FDR of 0.05 was chosen as the rank criterion, and 1000 simulations were run.

### Genetic alteration analysis

The traits of *SHOX2* genetic alterations were examined via the cBioPortal online dataset (https://www.cbioportal.org/) [13]. In the “Cancer Types Summary” module, we obtained the alteration frequency, mutation type and copy number alteration (CNA) data via TCGA database. A lollipop plot displayed the mutation distribution in SHOX2 protein via cBioPortal database. Moreover, databases of somatic mutations were obtained from TCGA datasets. The patients were divided into the first 25% SHOX2^high^ (*n* = 175) and the last 25% SHOX2^low^ (*n* = 175) groups according to the expression value of SHOX2. The maftools package was used in R software (https://www.r-project.org/) to visualize the somatic mutations of patients with 25% SHOX2^high^ and 25% SHOX2^low^ glioma.

### Tumor infiltration immune cells in pan-cancers

The correlations between tumor infiltration immune cells (TIICs) and SHOX2 mRNA expression in pan-cancers were investigated by the SangerBox portal, including B cells, CD8 + T cells, CD4 + T cells, eosinophil, gamma delta T cell, neutrophils, macrophages. Then, SangerBox website was applied to investigate the relationships between SHOX2 mRNA expression level and tumor mutation burden (TMB), microsatellite instability (MSI), neoantigens and immune checkpoint (ICP) in multiple cancers through TCGA datasets. The immune checkpoint genes included ADORA2A, BTNL2, CD200, CD244, CD274, CD86, CTLA4, HAVCR2, HHLA2, ICOSLG, IDO1, PDCD1, TIGIT, TNFRSF18, VTCN1. ImmuCellAI (http://bioinfo.life.hust.edu.cn/ImmuCellAI#!/) was used to explore the correlation between SHOX2 expression and TIICs in glioma. Moreover, TIMER2 portal was chosen to analyze the associations between SHOX2 expression and TIICs in LGG and GBM, including B cell, CD4 + T cells, CD8 + T cells, neutrophil, macrophage and dendritic cell. In TIMER2, we further investigated the correlations between SHOX2 mRNA expression level and CD4 + T cells or myeloid dendritic cell in pan-cancers, especially in the LGG via different algorithms, such as EPIC, TIMER, XCELL and CIBERSORT. In addition, the relationships between SHOX2 expression and stromal score, immune score and ESTIMATE score in LGG and GBM were explored via the SangerBox.

### Cell lines and culture

U-251MG (RRID: CVCL_0021) and LN-229 (RRID: CVCL_0393) human glioma cell lines were purchased from the Shanghai Cell Bank of the Chinese Academy of Sciences (Shanghai, China). A comprehensive cell line authentication service was used, and mycoplasma was periodically checked as well.

The DMEM Medium (Dulbecco’s Modified Eagle Medium, Gibco, USA) with 10% FBS (FBS, BI serum, Israel) were used to cultivate the glioma cell lines and cells incubated at 37 °C with 5% CO_2_.

### SiRNAs transfection and RNA isolation

The siRNAs using to silence the expression of SHOX2 were synthesized by Shanghai GenePharma Co., Ltd. The Lipofectamine ^TM^ RNAiMax (13778150, Thermo Fisher Scientific, USA) reagent was used to deliver siRNAs. In 6-well plates, 2 × 10^5^ cells were seeded each well. After 24 h, 40 pmol siRNA and 5 µL Lipofectamine ^TM^ RNAiMax were mixed each well for 10 min and mixture was added into the cells for transfection. Then, the cells were replenished with complete medium after 24 h, and TRIzol reagent (Thermo Fisher Scientific, Inc.) was used to lysis the cells after 48 h. The siRNA sequences were displayed: si-SHOX2-1, 5’-AACUUUGUUCGUGUGUAUCAATT-3’sense and 5’-GAUACACACGAACAAAGUUUATT-3’ antisense; si-SHOX2-2, 5’- AGAACAAAUAGUUACAAAGCUTT-3’sense and 5’- CUUUGUAACUAUUUGUUCUCCTT-3’ antisense; negative control (NC), 5’-UUCUCCGAACGUGUCACGUTT-3’sense and 5’-ACGUGACACGUUCGGAGAATT-3’ antisense.

### Reverse transcription (RT) and quantitative real-time polymerase chain reaction (qPCR)

RT-qPCR was used to examine the efficiency of SHOX2 knockdown. The cDNA was synthesized via GoScript reverse transcription system (Promega Corporation) and SHOX2 mRNA expression levels were detected via GoTaq®qPCR Master Mix (Promega Corporation) by ABI QuantStudio 3. As an internal control, GAPDH was applied. The PCR program was shown: 95˚C for 3 min, amplification with 40 cycles (15 sec at 95˚C and 60 sec at 60˚C) and a extension step (72˚C for 2 min). Every PCR experiment was carried out in triplicate. The primers used to qPCR were as follows: GAPDH, 5’-GGTGGTCTCCTCTGACTTCAACA-3’ forward and 5’-GTTGCTGTAGCCAAA TTCGTTGT-3’ reverse; SHOX2, 5’- GCGACTGACGGAGGTGTC-3’ forward and 5’- TCCAGGGTGAAATTGGTCCG-3’ reverse.

### CCK-8 experiment

Firstly, glioma cells (2 × 10^3^) were implanted into 96-well plates. After 24 h, siRNAs were used to silence the expression level of SHOX2 and CCK-8 reagent (APE x BIO, USA) was used to measure absorbance (OD value: 450 wavelength) lasted for 6 consecutive days (0, 1, 2, 3, 4, 5 and 6 days) (Serum-free medium: CCK8 reagent = 90 µL: 10µL per well). After incubation for 1 h at 37 ℃, OD value was detected on an enzyme-labeled instrument at 450 wavelength units.

### Wound healing and transwell experiments

In a 6-well plate, 2.5 × 10^5^ U-251MG or LN-229 cells were seeded per well. And si-NC, si-SHOX2-1 and si-SHOX2-2 were transfected into the 6-well plate. The cells in the plate were scratched with a 200µL pipetting head after 24 h, and fresh serum-free media was added in order to take pictures via an inverted microscope (IX81, Olympus Company, Japan) at 0, 12, and 24 h.

Transwell assay: we used the 8-µm pore filters coated with Matrigel glue (Corning, USA) to conduct this assay. The 6 × 10^4^ PANC1 and BXPC3 cells with 200 µL serum-free DMEM were seeded into the upper chamber and 500 µL DMEM with 10% FBS medium was added to the lower chamber. Cells were fixed with 4% paraformaldehyde and stained with 1% crystal violet after 24 h. The images were obtained using a microscope (Olympus, Japan).

### Colony formation assay

The cells were plated at a density of 6 × 10^3^ cells/well in 6-well plates. Cells were transfected with si-SHOX2-1, si-SHOX2-2 and si-NC after 24 h. Then, cells were cultured for 15 days. Further, 0.1% crystal violet was used to stain clones, and cells were photographed. The ImageJ was used to count the number of colonies.

### Statistical analysis

Using these online tools, statistical analyses were voluntarily calculated. Comparisons between multiple groups were made through one-way ANOVA and comparisons between CCK-8 groups were made via two-way ANOVA by Bonferroni’s post-hoc test. The significance levels for these findings were *P < 0.05, **P < 0.01, ***P < 0.001 and ****P < 0.0001.

## Results

### SHOX2 expression level is increased in pan-cancers including glioma

**Fig. 1:**
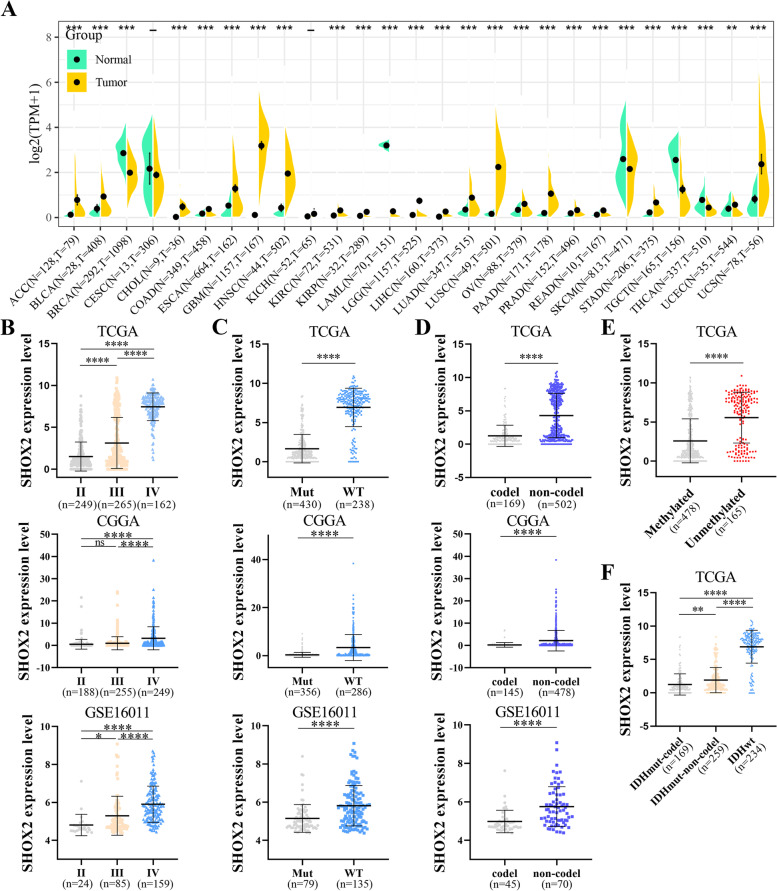
SHOX2 expression levels in normal tissues and tumors including glioma. **A** The differences of SHOX2 expression levels in 27 tumor tissues and normal tissues in TCGA combined with GTEx database by SangerBox. **B** The correlation between SHOX2 expression and glioma WHO grade (II, II and IV) in TCGA, CGGA and GSE16011 datasets. The expression level of SHOX2 in different IDH status (**C**) and 1p/19q status (**D**) of gliomas in TCGA, CGGA and GSE16011 datasets. **E** The expression level of SHOX2 in different MGMT promotor status of gliomas in TCGA database. **F** The correlation between SHOX2 expression and glioma three types (oligodendroglioma, astrocytoma and glioblastoma) in TCGA database. **P < 0.01, ***P < 0.001, ****P < 0.0001

Comparing the SHOX2 mRNA expression in tumor tissues and respective normal tissues to understand and analyze the differences, the Cancer Genome Atlas (TCGA) and Genotype-Tissue Expression (GTEx) expression profiles for several cancers or normal tissues were studied to determine whether SHOX2 mRNA expression in normal tissues differed from that in tumor tissues. The SHOX2 mRNA expression levels in all TCGA tumor datasets are shown in Fig. 1A. SHOX2 mRNA expressions were upregulated in multiple tumors compared with the corresponding normal tissues. Analyses suggested that the mRNA expression level of SHOX2 was significantly increased in 20 cancers by TCGA and GTEx databases, including adrenocortical carcinoma (ACC), bladder urothelial carcinoma (BLCA), colon adenocarcinoma (COAD), cholangiocarcinoma (CHOL), esophageal carcinoma (ESCA), glioblastoma multiforme (GBM), head and neck cancer (HNSC), kidney renal papillary cell carcinoma (KIRP), kidney renal clear cell carcinoma (KIRC), brain lower grade glioma (LGG), liver hepatocellular carcinoma (LIHC), lung adenocarcinoma (LUAD), lung squamous cell carcinoma (LUSC), ovarian serous cystadenocarcinoma (OV), prostate adenocarcinoma (PRAD), pancreatic adenocarcinoma (PAAD), rectum adenocarcinoma (READ), stomach adenocarcinoma (STAD), uterine corpus endometrial carcinoma (UCEC) and uterine carcinosarcoma (UCS) (Fig. 1A). However, SHOX2 mRNA expression was lower in breast invasive carcinoma (BRCA), acute myeloid leukemia (LAML), testicular germ cell tumors (TGCT), skin cutaneous melanoma (SKCM) and thyroid carcinoma (THCA) (Fig. 1A).

Then, SHOX2 mRNA expression were analyzed in WHO grades and new types of gliomas. Figure 1B showed that the expression levels of SHOX2 were positively related to grades of glioma patients in TCGA, CGGA and GSE16011 databases. The expression of SHOX2 in glioma patients with higher WHO grade is higher in TCGA, CGGA and GSE16011 databases. (Fig. 1B). Based on the World Health Organization’s (WHO) classification of tumors of the fifth edition of the central nervous system (CNS) (WHO CNS5), gliomas can be classified into three types: isocitrate dehydrogenase (IDH)-mutant (mut + non-codel) astrocytoma, IDH-mutant and 1p/19q-codeleted (mut + codel) oligodendroglioma, and IDH-wildtype (IDH-wild) glioblastoma [14]. Better survival rates for glioma patients are associated with IDH mutations and chromosomal 1p/19q codeletions [15]. For glioblastoma patients receiving the treatment of temozolomide (TMZ), the O6-methylguanine DNA methyltransferase (MGMT) promoter methylation status is a prognostic predictor [16]. Moreover, we investigated the correlations between SHOX2 mRNA expression and the status of IDH gene mutations, 1p/19q codeletion or MGMT promoter methylation. Figure 1C showed that the SHOX2 mRNA expression was upregulated in patients with IDH-WT compared to patients with IDH-mutant in TCGA, CGGA and GSE16011 databases. The mRNA expression level of SHOX2 was upregulated in patients with 1p/19q-noncodel compared to patients with 1p/19q-codel in TCGA, CGGA and GSE16011 databases (Fig. 1D). In addition, TCGA database showed that mRNA expression level of SHOX2 was upregulated in patients with MGMT unmethylated compared to patients with MGMT methylated (Fig. 1E). The expression levels of SHOX2 mRNA were associated with the WHO CNS5 types. TCGA database also displayed that SHOX2 mRNA levels in the glioma patients with wild-type IDH were higher compared with patients of IDH-mut and 1p/19q non-codeletion or IDH-mut and 1p/19q codeletion (Fig. 1F). A summary of our results suggested that SHOX2 mRNA expression levels were increased in multiple tumors, including glioma. The expression level of SHOX2 was related to the grades and clinical features of glioma patients.

### SHOX2 expression is related to prognosis in multiple cancers, including glioma

**Fig. 2:**
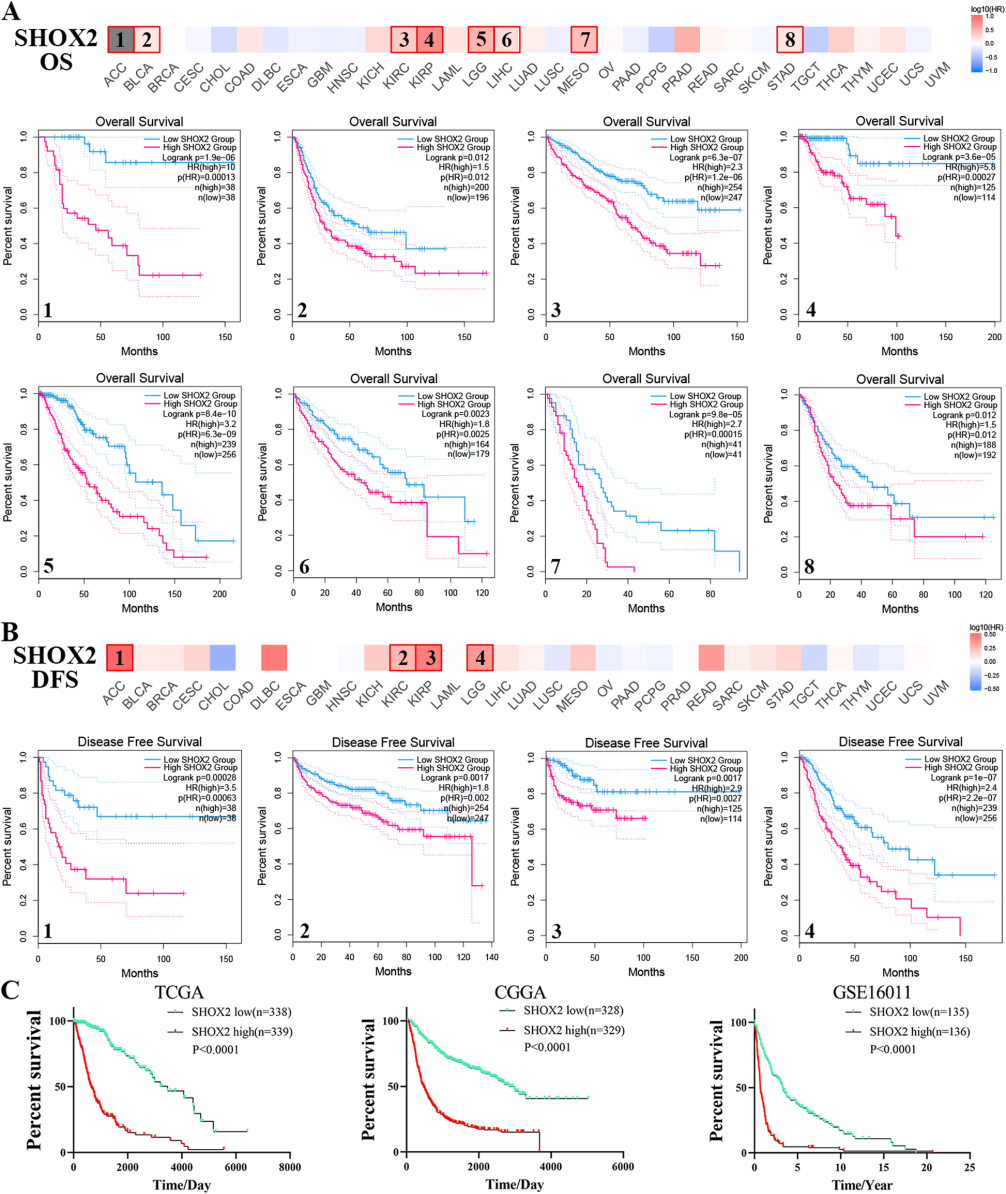
The Correlation between SHOX2 expression and survival prognosis in various types of cancer based TCGA database. **A** Survival maps of overall survival (OS) and disease-free survival (DFS) (**B**) between high and low SHOX2 expression in different types of cancer compared using a log-rank test via GEPIA2. **C** The correlation between SHOX2 expression and prognosis of glioma patients in TCGA, CGGA and GSE16011 datasets

In order to investigate the correlation between SHOX2 mRNA expression and prognosis in a variety of cancers, GEPIA2 and Sangerbox portals were used. Based on survival-related information obtained from the TCGA, the correlation between SHOX2 mRNA levels and prognosis was explored and the OS or DFS curves were plotted. There was a poorer prognosis and shorter overall survival (OS) rate among multiple tumor patients with high levels of SHOX2 mRNA expression, including ACC, BLCA, KIRC, KIRP, LGG, LIHC, MESO and STAD (Fig. 2A). In addition, there was a poorer prognosis and shorter disease-free survival (DFS) rate among patients with multiple tumors who had high levels of SHOX2 mRNA expression, including ACC, KIRC, KIRP and LGG (Fig. 2B). In TCGA, CGGA and GSE16011 databases, there was a poorer prognosis among patients with high levels of SHOX2 mRNA expression compared to patients with low SHOX2 mRNA expression (Fig. 2C).

Then, Cox regression analysis was applied to further explore the relationships between SHOX2 mRNA levels and OS in these tumors. These results suggested that high mRNA expression levels of SHOX2 were related to a worse OS in the GBMLGG (HR = 1.33), LGG (HR = 1.27), KIPAN (HR = 1.28), KIRP (HR = 1.31), KIRC (HR = 1.24), MESO (HR = 1.29), ACC (HR = 1.34), LAML (HR = 1.12), BLCA (HR = 1.12), LIHC (HR = 1.12), LUAD (HR = 1.09), STAD (HR = 1.11), THCA (HR = 1.48) and UCEC (HR = 1.20) (Fig. S1A). Moreover, Kaplan-Meier plotter portal was used to analyze the relationship between SHOX2 mRNA expression and prognosis. The results showed that high SHOX2 expression levels were significantly correlated with shorter OS in gastric cancer (GC), liver cancer, lung cancer and ovarian cancer (OV) via Kaplan-Meier plotter portal. Overall, the analyses showed that high levels of SHOX2 mRNA expression were correlated with a poorer prognosis in pan-cancers, including in glioma (Especially in the LGG) (Fig. S1B).

### SHOX2 and its related genes in pan-cancers, including glioma

**Fig. 3:**
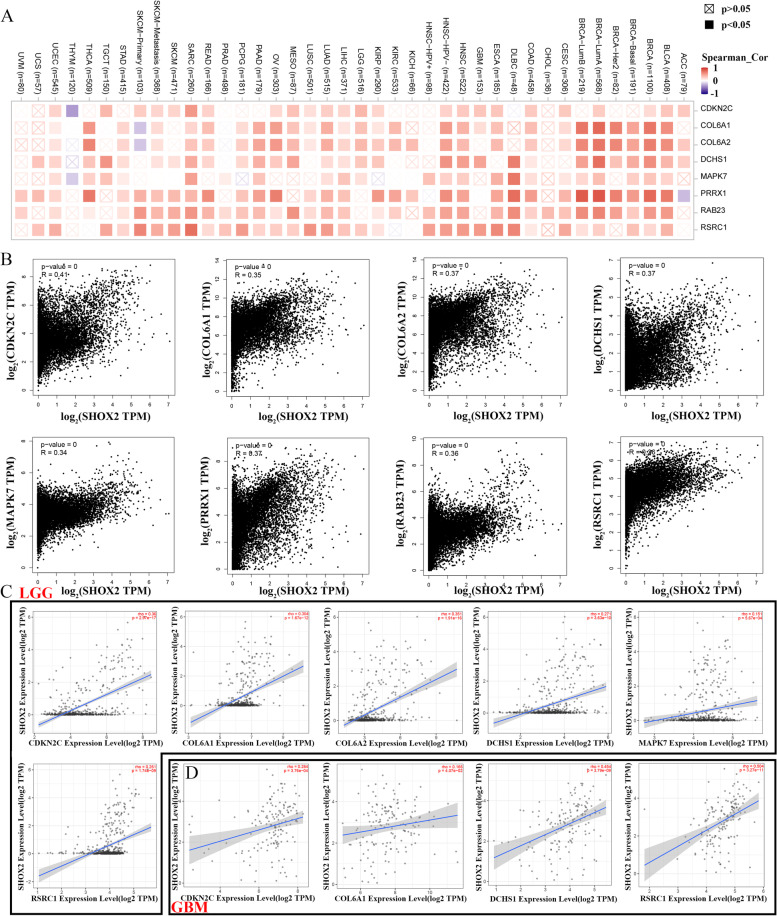
SHOX2-related genes analyses in pan-cancers including glioma. **A** The corresponding heatmap of top 8 SHOX2-related genes in pan-cancers via TIMER2. **B** The correlation analyses between SHOX2 and the top 8 SHOX2-related genes in pan-cancers by TCGA database. **C** The correlation analyses between SHOX2 and the SHOX2-related genes in LGG by TIMER2. **D** The correlation analyses between SHOX2 and the SHOX2-related genes in GBM by TIMER2. The partial correlation (cor) and *P*-value was generated via the purity-adjusted Spearman’s rank correlation test

**Fig. 4:**
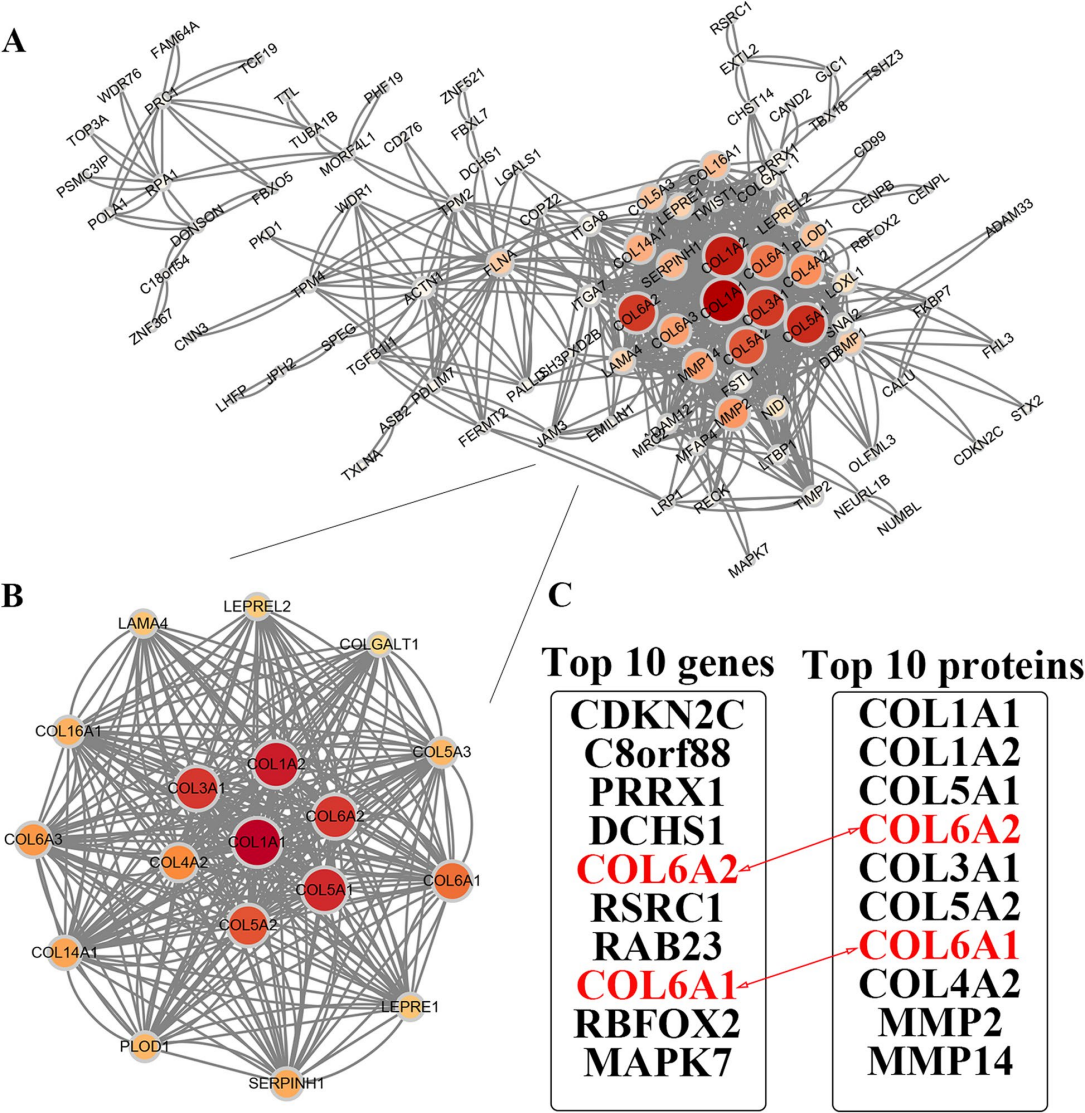
Analysis of SHOX2-related genes protein interaction. **A** Protein network interaction analysis of top 200 SHOX2-related genes via STRING portal. **B** Key networks in protein network interaction analysis of top 200 SHOX2-related genes. **C** The intersection of top10 SHOX2-related genes and top 10 SHOX2-related gene network proteins

Using the GEPIA2 online tool, we examined the SHOX2-related genes to identify the crucial role of the *SHOX2* gene in tumor pathogenesis. We acquired the top 8 genes that positively related to SHOX2 expression by the GEPIA2 portal and these top 8 genes, including CDKN2C, COL6A1, COL6A2, DCHS1, MAPK7, PRRX1, RAB23 and RSRC1, were drawn into heatmap in pan-cancers via TIMER2 (Fig. 3A). In pan-cancer analysis, SHOX2 expression positive correlated with CDKN2C (*R* = 0.41), COL6A1 (*R* = 0.35), COL6A2 (*R* = 0.37), DCHS1 (*R* = 0.37), MAPK7 (*R* = 0.34), PRRX1 (*R* = 0.37), RAB23 (*R* = 0.36) and RSRC1 (*R* = 0.36) via GEPIA2 (Fig. 3B). Moreover, we analyzed the correlations between SHOX2 expression and these top 8 related genes in LGG and GBM. The results showed that SHOX2 expression was positively associated with CDKN2C, COL6A1, COL6A2, DCHS1, MAPK7 and RSRC1 in LGG (Fig. 3C) and positively associated with CDKN2C, COL6A1, DCHS1 and RSRC1 in GBM (Fig. 3D) via TIMER2. Then, we analyzed top 200 SHOX2-related genes in STRING portal, and assigned different nodes with different sizes according to the number of node networks (Fig. 4A). The nodes with more networks were more important. We identified a key protein interaction network of SHOX2-related genes (Fig. 4B). We obtained COL6A2 and COL6A1 in the interaction of the top 10 SHOX2-related genes and the top 10 proteins of SHOX2-related genes protein network, indicating that the important function of SHOX2 in tumors may be realized via influencing COL6A2 and COL6A1 expression (Fig. 4C).

Furthermore, to further studying the biological functions of SHOX2 in glioma (LGG and GBM). We analyzed the top 50 genes in glioma via LinkedOmics online database, which were positively or negatively correlated with SHOX2, as shown in the heat map (Fig. S2A). Then, Fig. S2B displayed the Kyoto Encyclopedia of Genes and Genomes (KEGG) enrichment analysis of SHOX2-related genes in glioma. The top 50 genes positively correlated with SHOX2 could be enriched in vibrio cholerae infection, thyroid hormone synthesis, cell cycle, hippo signaling pathway, mismatch repair, ferroptosis, DNA replication, P53 signaling pathway and so on. The top 50 genes negatively correlated with SHOX2 could be enriched in synaptic vesicle cycle, circadian rhythm, hedgehog signaling pathway, fatty and metabolism (Fig. S2B). The results demonstrated that SHOX2 could regulate metabolism and DNA repair associated signaling pathways in glioma. These results suggested that SHOX2 expression was related to cell cycle, metabolism or DNA damage repair.

### Alterations in the *SHOX2* gene in multiple cancers

**Fig. 5:**
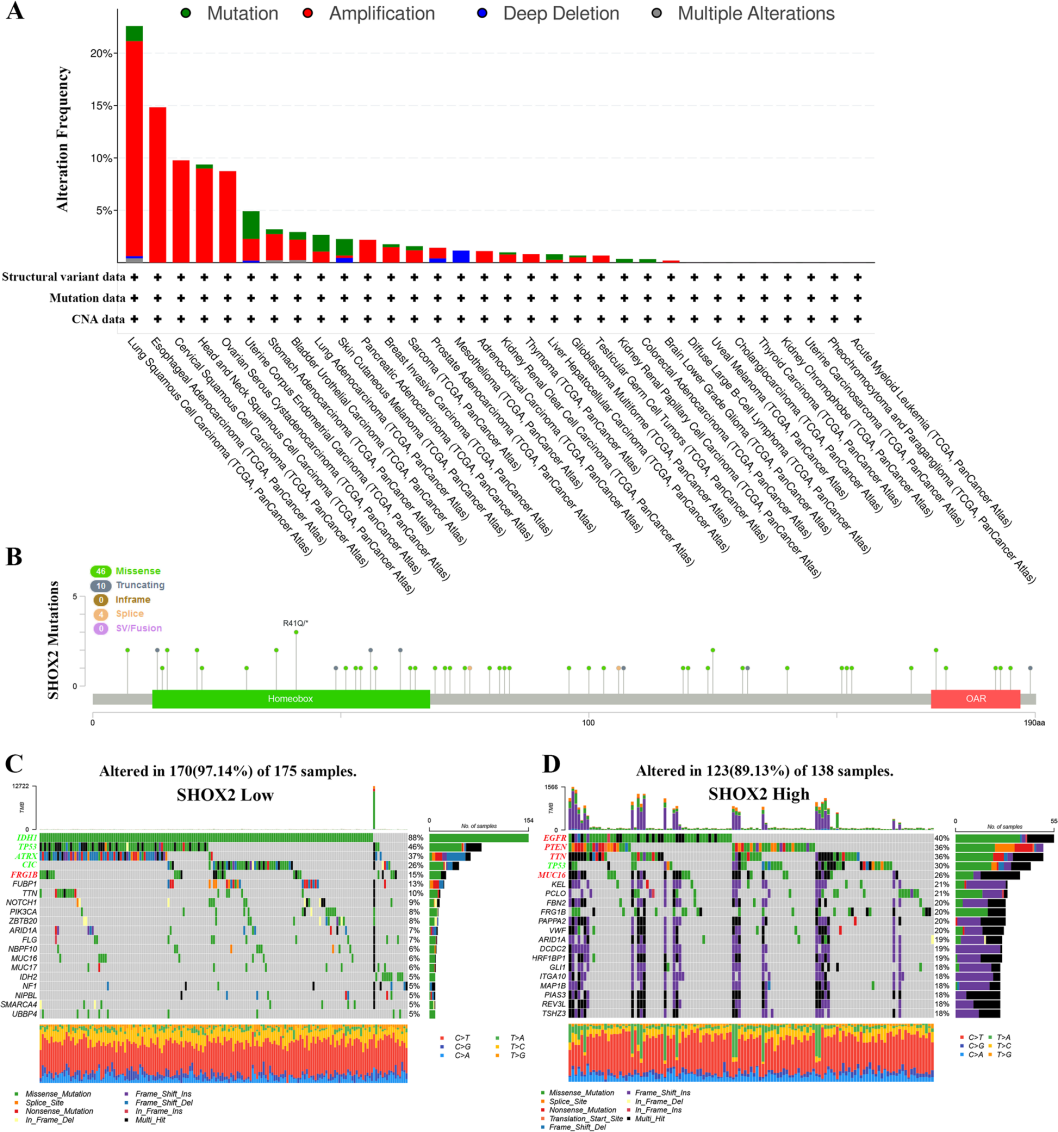
Mutation feature of SHOX2 in different tumors. The alteration frequency with mutation type (**A**) and mutation site (**B**) are showed. **C** Detection of differential somatic mutations in gliomas, including 25% SHOX2^low^ group (**C**) and 25% SHOX2^high^ group (**D**)

Oncogene or tumor suppressor gene mutations, deletions, or amplifications are related to the development and progression of tumors [17]. Therefore, utilizing the cBioPortal portal, we first examined various sorts of modifications in the *SHOX2* gene, such as mutations, structural variants, amplifications, and deep deletions. The most common genetic alteration in the *SHOX2* gene were amplifications in the lung squamous cell carcinoma (LUSC) (20.53%), esophageal carcinoma (ESCA) (14.84%), cervical squamous cell carcinoma (CESC) (9.76%), head and neck cancer (HNSC) (8.99%) and ovarian serous cystadenocarcinoma (OV) (8.73%); mutations (2.65%) in the uterine corpus endometrial carcinoma (UCEC); and deep deletions (1.15%) in the Mesothelioma (MESO) (Fig. 5A). Secondly, we studied the genetic alterations within SHOX2 across a variety of tumors sequentially. The results indicated that the primary type of genetic alteration of SHOX2 was missense mutation (Fig. 5B). Then, the association between SHOX2 expression and somatic mutations was analyzed in the TCGA glioma dataset. The SHOX2^high^ group (*n* = 175) showed high frequency of somatic mutations in the *EGFR* (40%), *PTEN* (36%), *TTN* (36%) and *MUC16* (26%) genes in glioma (Fig. 5D). The SHOX2^low^ group (*n* = 175) showed high frequency of mutations in the *IDH1* (88%), *TP53* (46%), *ATRX* (37%), and *CIC* (26%) genes in glioma (Fig. 5C). This finding suggested that SHOX2 alterations may contribute to the progression of cancer.

### SHOX2 regulates tumor infiltration of immune cells in multiple cancers including glioma

**Fig. 6:**
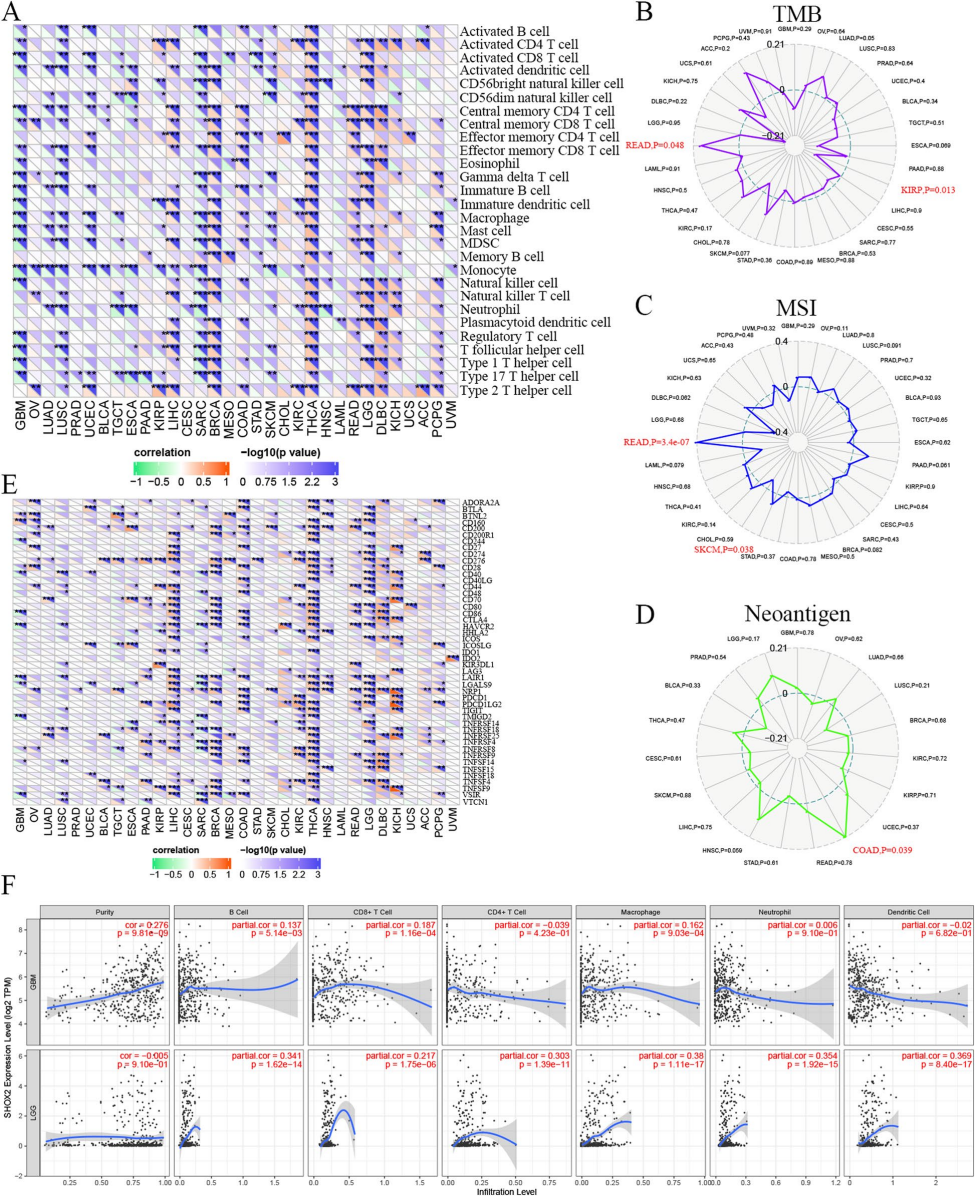
Correlation analyses between SHOX2 expression and immune infiltration levels. **A** The relationships between SHOX2 expression and immune cell infiltration level in various types of cancer via Sangerbox. The correlations between SHOX2 expression and TMB (**B**), MSI (**C**), neoantigen (**D**) and ICP-related genes (**E**) in multiple cancers via Sangerbox. **F** The relationships between SHOX2 expression and B cell, CD8 + T cell, CD4 + T cell, macrophage, neutrophil and dendritic cell in LGG or GBM via Sangerbox. *P < 0.05, **P < 0.01, ***P < 0.001

Numerous literatures have reported that tumor infiltrating immune cells (TIICs) played a key role in tumorigenesis and progression [18, 19]. TIICs are important components of the tumor immune microenvironment (TIM) and play a critical role in the initiation, metastasis, and progression of tumors [18]. To investigate whether SHOX2 expression affects the levels of TIICs, we analyzed the relationship between SHOX2 expression levels and the composition of TIICs in various tumors. Firstly, we explored the correlation between TIIC levels and SHOX2 expression in multiple cancers via the Sangerbox portal. The results revealed that SHOX2 expression negative correlated with multiple immune cell types in GBM, LUAD, LUSC, UCEC and SARC, and positive correlated with immune cell types in LIHC, BRCA, THCA, READ, LGG and DLBC (Fig. 6A). The immune cell types included activated CD8 + and CD4 + T cells, CD8 + T cells and central memory CD4+, gamma delta T cells, activated dendritic cells, effector memory CD4 + and CD8 + T cells, natural killer cells, immature B cells, MDSCs, memory B cells, macrophages, regulatory T cells and natural killer T cells (Fig. 6A).

Antitumor immunity is a strong indicator of tumor immunotherapy efficacy and is correlated with tumor mutation burden (TMB), neoantigens, and microsatellite instability (MSI) [20]. Immune checkpoint inhibitors are effective against high MSI (MSI-H) and TMB tumors and mutated antigens specifically expressed by the tumor, known as neoantigens, are promising targets for tumor immunotherapy employing T cell [21, 22]. Secondly, we explored the correlation between SHOX2 expression levels and MSI, TMB or neoantigens to verify if SHOX2 was a predictor of immunotherapeutic responses in pan-cancers. SHOX2 expression displayed positive correlation with TMB in READ (*P* = 0.048) and negative association with TMB in KIRP (*P* = 0.013) (Fig. 6B). SHOX2 expression showed positive correlation with MSI in READ (*P* = 3.4e-07) and negative association in SKCM (*P* = 0.038) (Fig. 6C). SHOX2 expression showed positive relationship with neoantigens in COAD (*P* = 0.039) (Fig. 6D). In cancer immunotherapeutic treatments, immune checkpoint (ICP) blockade proteins are potential targets as they regulate immune cell infiltration into tumor microenvironment [23]. Then, we investigated the correlations between ICP expression levels and SHOX2 expression in multiple tumors. SHOX2 expression showed a positive correlation with ICP genes in LIHC, BRCA, COAD, THCA, LGG, DLBC and KICH (Fig. 6E). SHOXA2 expression showed positive relation with 36 out of 47 ICP genes in THCA and 32 out of 47 ICP genes in LIHC (Fig. 6E). In LGG, SHOXA2 expression showed positive relation with 29 out of 47 ICP genes. These results indicated that SHOX2 affected the sensitivity of LIHC, BRCA, COAD, THCA, LGG, DLBC and KICH to the immune checkpoint inhibitor therapies. In GBM and SARC, SHOX2 expressions showed negative relation with the ICP genes, indicating that patients with high SHOX2 expression might respond poorly to immunotherapies targeting ICP genes in GBM and SARC. In glioma, we analyzed the correlation between SHOX2 expression level and ICP genes (Ligands and receptors), and the results showed that SHOX2 expression was positively correlated with many ligands and receptors, such as CD274, PDCD1LG2, BTLA, CD86, ICOSLG, CD70, PVR, CD40LG, CD244, CD226, HAVCR2, CD27, CD40, CD96, CD200R1, TNFRSF9, ICOS, PDCD1, CTLA4 and TNFRSF14 (Fig. S3).

**Fig. 7:**
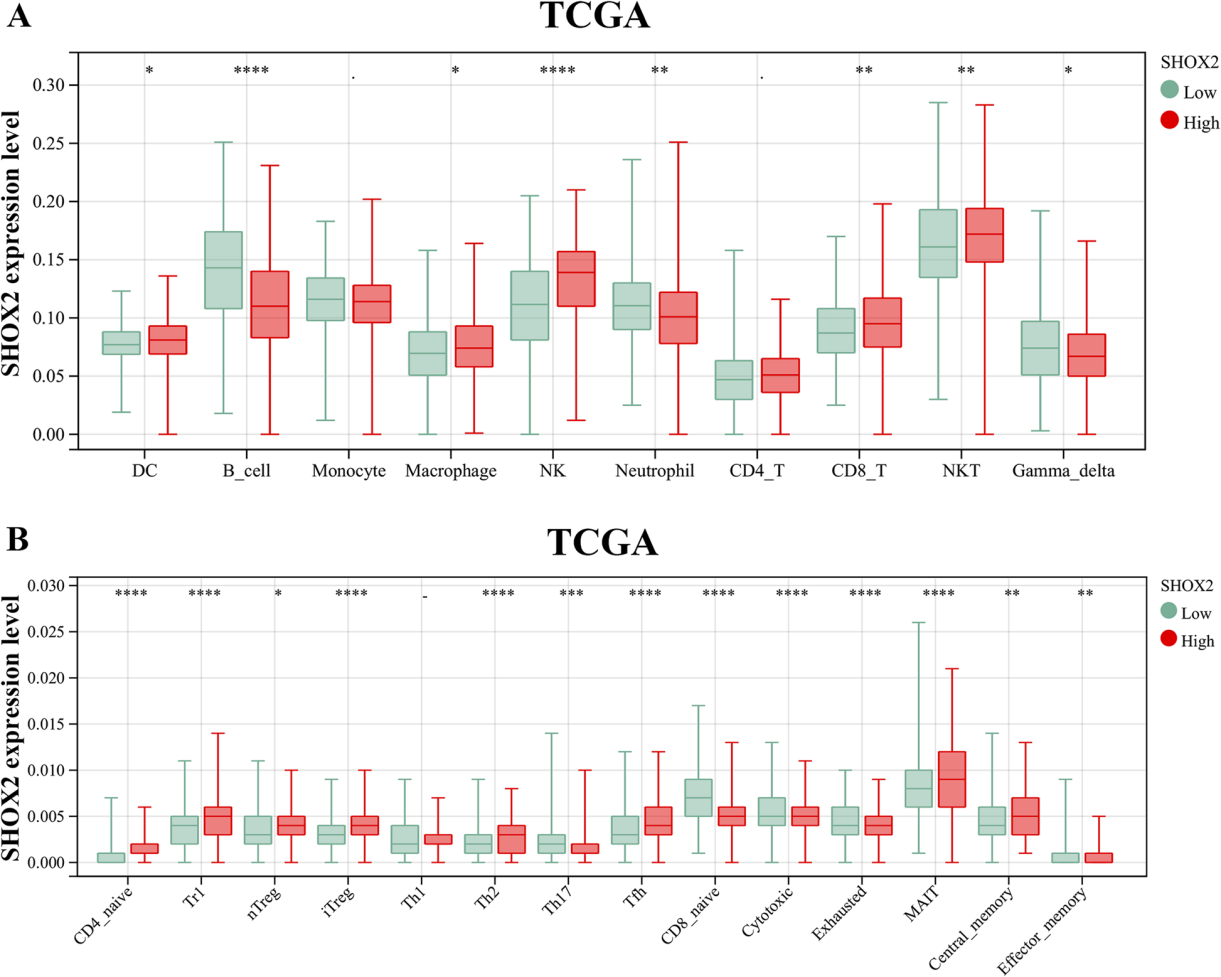
The correlations between SHOX2 expression and TIICs via ImmuCellAI in TCGA database

We further verified the relationship between SHOX2 expression and TIICs in glioma via TIMER2. The results showed that SHOX2 expression level was positively related to CD8 + T cell, B cell and macrophage in GBM and positively correlated with B cell, CD8 + T cell, CD4 + T cell, macrophage, neutrophil and dendritic cell in LGG (Fig. 6F). Then, we analyzed the correlation between SHOX2 expression and TIICs in glioma via ImmuCellAI in TCGA and CGGA databases. In TCGA, SHOX2 expression was positively related to DC, macrophage, NK, CD8_T, NKT, CD4_naive, Tr1, nTreg, Th2, Tfh, MAIT and Central_memory cells and negatively related to B_cell, neutrophil, gamma_delta, Th17, CD8_naïve, cytotoxic, exhausted and effector_memory cells (Fig. 7A-B). In CGGA cohort, SHOX2 expression was positively related to macrophage, NK, CD4_naive, Tr1, nTreg and iTreg cells and negatively related to B_cell, CD8_T, Th1, Th2, CD8_naive, cytotoxic and central_memory cells (Fig. S4).

**Fig. 8:**
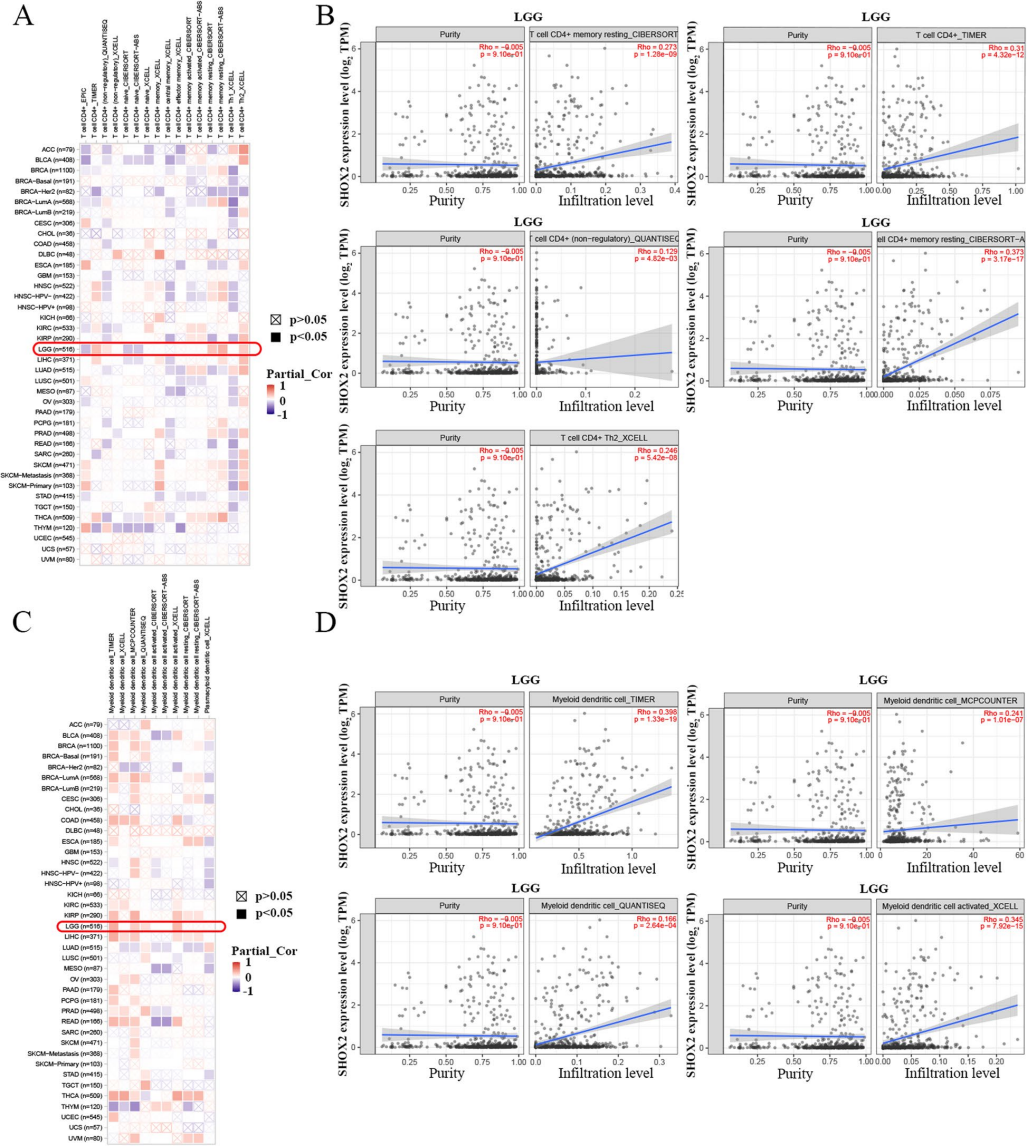
The correlations between SHOX2 expression and CD4 + T cell or myeloid dendritic cell in multiple cancers including LGG. **A** The correlations between SHOX2 expression and CD4 + T cell in multiple cancers. **B** The correlations between SHOX2 expression and CD4 + T cell in LGG via different algorithms. **C** The relationships between SHOX2 expression and myeloid dendritic cell in multiple cancers. **D** The relationships between SHOX2 expression and myeloid dendritic cell in LGG via different algorithms

Moreover, the correlations between SHOX2 expression and CD4 + T cell (Fig. 8A) and myeloid dendritic cell (Fig. 8C) infiltrating levels were analyzed via TIMER2 in multiple tumors. In LGG, the expression level of SHOX2 was positively associated with the infiltration level of CD4 + T cell obtained by the five algorithms (Fig. 8B) and positively correlated with the infiltration level of myeloid dendritic cell obtained by the four algorithms (Fig. 8D). Furthermore, we evaluated the association between SHOX2 expression levels and ESTIMATE scores (stromal, immune, and ESTIMATE scores) in LGG and GBM. Stromal score reflects the proportion of stromal cells in the tumor tissues; immune score reflects the proportion of infiltrated immune cells in the tumor tissues; and ESTIMATE score is the sum of stromal and immune scores, and reflects the status of the TIM and tumor purity. Our results showed negative relationship between SHOX2 expression and the stromal, immune and ESTIMATE scores in GBM and positive correlation between SHOX2 expression and the stromal, immune and ESTIMATE scores in LGG (Fig. S5A-C). This indicated that high expression level of SHOX2 was related to decreased infiltration of stromal and immune cells in GBM, thereby resulting in high tumor purity. These results suggested that LGG, but not GBM, may be able to benefit from immunotherapy targeted SHOX2.

As a result, SHOX2 mediated the regulation of ICP genes and was a promising target for immunotherapy of tumors, including LGG.

### SHOX2 can promote proliferation, migration and invasion of glioma cells in vitro

**Fig. 9:**
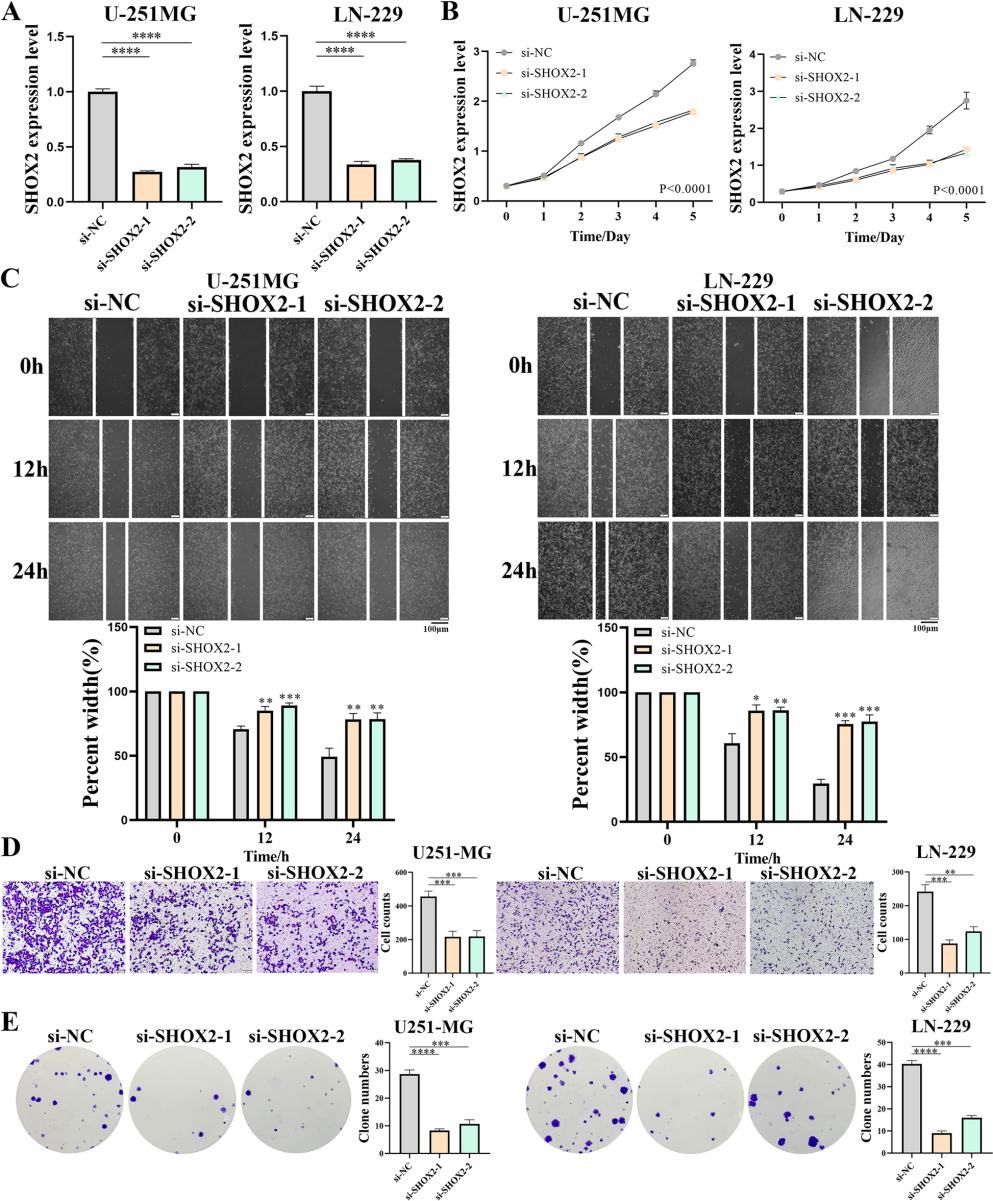
The biological functions of SHOX2 in glioma cells. **A** MRNA expression levels of SHOX2 were analyzed following SHOX2 siRNAs knockdown via RT-qPCR. **B** CCK-8 assay was used to measure cell proliferation following knockdown by SHOX2 siRNAs in U-251MG and LN-229 cells. **C** Wound healing assay was used to measure cell migration following knockdown by SHOX2 siRNAs in U-251MG and LN-229 cells. **D** Transwell assay used to measure cell invasion following knockdown by SHOX2 siRNAs in U-251MG and LN-229 cells. **E** Colony formation assay performed using U-251MG and LN-229 cells following knockdown via SHOX2 siRNAs. *P < 0.05, **P < 0.01, ***P < 0.001, ****P < 0.0001

Next, U-251MG and LN-229 glioma cell lines were applied to verify the biological functions of SHOX2. SHOX2-targeting siRNAs (si-SHOX2-1 and si-SHOX2-2) specific to silence the mRNA expression levels of SHOX2 were used in the U-251MG and LN-229 cells (Fig. 9A). CCK-8 assay suggested that SHOX2 knockdown suppressed the cell proliferation of U-251MG and LN-229 cells in vitro (Fig. 9B). In addition, wound healing and Transwell experiments indicated that SHOX2 knockdown suppressed the cell migration and invasion of U-251MG and LN-229 cells in vitro (Fig. 9C-D). In colony formation assay, reduced SHOX2 expression impaired the colony formation capacities of glioma cells (Fig. 9E). These results revealed that silencing of SHOX2 expression remarkable reduced the proliferation, migration, invasion and colony formation capacities of U-251MG and LN-229 cells in vitro. These results showed that SHOX2 could promote proliferation, migration and invasion of glioma cells in vitro.

## Discussion

SHOX2, a significant transcriptional regulator in multiple genetic disorders, has been verified to be a powerful biomarker for the evaluation and diagnosis of a wide variety of cancers, including lung cancer [24, 25]. Yang et al. found that high SHOX2 expression was associated with tumor recurrence in hepatocellular carcinoma [6]. In this research, we investigated the biological functions of SHOX2 in a variety of cancers. In addition, studies reported that SHOX2 expression had also been shown to be an independent prognostic indicator in grade II and III diffuse gliomas [26]. Therefore, while studying the biological functions of SHOX2 in pan-cancers, we further studied the biological function of SHOX2 in glioma.

The present study found that SHOX2 was increased in a variety of tumors. In our research, we first revealed that the SHOX2 mRNA expression were higher in various tumors compared with normal tissues, including ACC, BLCA, COAD, CHOL, ESCA, GBM, HNSC, KIRP, KIRC, LGG, LIHC, LUSC, LUAD, OV, PRAD, PAAD, READ, STAD, UCEC and UCS. The expression levels of SHOX2 were significantly associated with the grades and clinical features of glioma patients, such as IDH status, 1p/19q status, MGMT status and new types. Higher mRNA levels of SHOX2 were correlated with a worse OS in ACC, BLCA, KIRC, KIRP, LGG, LIHC, MESO and STAD, and a shorter DFS in ACC, KIRC, KIR and LGG. Cox regression analysis demonstrated that mRNA expression levels of SHOX2 were related to a shorter OS in the GBMLGG, LGG, KIPAN, KIRP, KIRC, MESO, ACC, LAML, BLCA, LIHC, LUAD, STAD, THCA and UCEC. Moreover, KEGG enrichment analyses of SHOX2-related genes revealed that the expression level of SHOX2 was significantly correlated with cell cycle, metabolism or DNA damage repair in glioma patients. And patients with tumors lacking SHOX2 alterations had a better prognosis of OS compared to patients with SHOX2 alterations, suggesting its oncogenic role in multiple tumors. Katja U Schneider’s research also verified this point [27].

Furthermore, as a significant component of the immune microenvironment, TIICs play a key role in the tumorigenesis, progression and treatment of tumors. The SHOX2 mRNA levels were significantly related to multiple TIICs in pan-cancers, including LGG and GBM. A positive correlation was observed between SHOX2 mRNA expression levels and TIICs in LGG and a negative correlation between SHOX2 mRNA expression levels and TIICs in GBM. In addition, SHOX2 affected the sensitivity of cancer patients to immunotherapy was next assessed. TMB, MSI, neoantigen and ICP analyses showed that SHOX2 might be a promising target for the treatment of some tumor patients, especially in immunotherapy. It seemed that LGG was more sensitive to immunotherapy than GBM. In vitro assays also showed that SHOX2 knockdown significantly reduced the proliferation, migration, invasion and colony formation capacities of U-251MG and LN-229 cells. Based on these results, it was hypothesized that SHOX2 promoted the growth of pan-cancers, particularly in glioma.

## Conclusion

In conclusion, these results suggested that SHOX2 may be a promising prognostic marker and a potential factor for predicting sensitivity to immunotherapy in patients with malignant tumors, particularly glioma.

## Supplementary Information

**Additional file 1: Supplementary Figure 1.** Correlation between SHOX2 mRNA expression and prognosis of pan-cancers. **Supplementary Figure 2.** KEGG enrichment analyses of SHOX2-related genes in glioma. **Supplementary Figure 3.** Correlations between SHOX2 expression and ICP genes in glioma. **Supplementary Figure 4.** The correlations between SHOX2 expression and TIICs via ImmuCellAI in CGGA database. **Supplementary Figure 5.** Analysis showed the correlation between SHOX2 expression and ESTIMATE score in glioma.

## Data Availability

The datasets used and/or analyzed during the present study are available from the corresponding author on reasonable request.

## Abbreviations

ACC: Adrenocortical carcinoma
BLCA: Bladder urothelial carcinoma
BRCA: Breast invasive carcinoma
CESC: Cervical squamous cell carcinoma and endocervical adenocarcinoma
CHOL: Cholangiocarcinoma
COAD: Colon adenocarcinoma
ESCA: Esophageal carcinoma
GBM: Glioblastoma multiforme
HGG: Brain higher grade glioma
HNSC: Head and neck cancer
KICH: Kidney chromophobe
KIRC: Kidney renal clear cell carcinoma
KIRP: Kidney renal papillary cell carcinoma
LAML: Acute myeloid leukemia
LGG: Brain lower grade glioma
LIHC: Liver hepatocellular carcinoma
LUAD: Lung adenocarcinoma
LUSC: Lung squamous cell carcinoma
OS: Overall survival
OV: Ovarian serous cystadenocarcinoma
PAAD: Pancreatic adenocarcinoma
PRAD: Prostate adenocarcinoma
READ: Rectum adenocarcinoma
SKCM: Skin cutaneous melanoma
STAD: Stomach adenocarcinoma
TGCT: Testicular germ cell tumors
THCA: Thyroid carcinoma
UCEC: Uterine corpus endometrial carcinoma
UCS: Uterine carcinosarcoma

## References

[1] Puskaric S, Schmitteckert S, Mori AD, Glaser A, Schneider KU, Bruneau BG, Blaschke RJ, Steinbeisser H, Rappold G (2010). Shox2 mediates Tbx5 activity by regulating Bmp4 in the pacemaker region of the developing heart. Hum Mol Genet.

[2] Espinoza-Lewis RA, Yu L, He F, Liu H, Tang R, Shi J, Sun X, Martin JF, Wang D, Yang J (2009). Shox2 is essential for the differentiation of cardiac pacemaker cells by repressing Nkx2-5. Dev Biol.

[3] Gopalakrishnan S, Van Emburgh BO, Robertson KD (2008). DNA methylation in development and human disease. Mutat Res.

[4] Zoghbi HY, Beaudet AL (2016). Epigenetics and human disease. Cold Spring Harb Perspect Biol.

[5] Shen C, Wang K, Deng X, Chen J (2022). DNA N(6)-methyldeoxyadenosine in mammals and human disease. Trends Genet.

[6] Yang T, Zhang H, Cai SY, Shen YN, Yuan SX, Yang GS, Wu MC, Lu JH, Shen F (2013). Elevated SHOX2 expression is associated with tumor recurrence of hepatocellular carcinoma. Ann Surg Oncol.

[7] Zhao J, Dong Y, Kang W, Go MY, Tong JH, Ng EK, Chiu PW, Cheng AS, To KF, Sung JJ (2014). Helicobacter pylori-induced STAT3 activation and signalling network in gastric cancer. Oncoscience.

[8] Teng Y, Loveless R, Benson EM, Sun L, Shull AY, Shay C (2021). SHOX2 cooperates with STAT3 to promote breast cancer metastasis through the transcriptional activation of WASF3. J Exp Clin Cancer Res.

[9] Hu J, Qiu D, Yu A, Hu J, Deng H, Li H, Yi Z, Chen J, Zu X (2021). YTHDF1 is a potential pan-cancer biomarker for prognosis and immunotherapy. Front Oncol.

[10] Tang Z, Kang B, Li C, Chen T, Zhang Z (2019). GEPIA2: an enhanced web server for large-scale expression profiling and interactive analysis. Nucleic Acids Res.

[11] Li T, Fu J, Zeng Z, Cohen D, Li J, Chen Q, Li B, Liu XS (2020). TIMER2.0 for analysis of tumor-infiltrating immune cells. Nucleic Acids Res.

[12] Vasaikar SV, Straub P, Wang J, Zhang B (2018). LinkedOmics: analyzing multi-omics data within and across 32 cancer types. Nucleic Acids Res.

[13] Wu P, Heins ZJ, Muller JT, Katsnelson L, de Bruijn I, Abeshouse AA, Schultz N, Fenyo D, Gao J (2019). Integration and analysis of CPTAC proteomics data in the context of cancer genomics in the cBioPortal. Mol Cell Proteomics.

[14] Louis DN, Perry A, Wesseling P, Brat DJ, Cree IA, Figarella-Branger D, Hawkins C, Ng HK, Pfister SM, Reifenberger G (2021). The 2021 WHO classification of tumors of the central nervous system: a summary. Neuro Oncol.

[15] Eckel-Passow JE, Lachance DH, Molinaro AM, Walsh KM, Decker PA, Sicotte H, Pekmezci M, Rice T, Kosel ML, Smirnov IV (2015). Glioma groups based on 1p/19q, IDH, and TERT promoter mutations in tumors. N Engl J Med.

[16] Chen X, Zhang M, Gan H, Wang H, Lee JH, Fang D, Kitange GJ, He L, Hu Z, Parney IF (2018). A novel enhancer regulates MGMT expression and promotes temozolomide resistance in glioblastoma. Nat Commun.

[17] Martincorena I, Raine KM, Gerstung M, Dawson KJ, Haase K, Van Loo P, Davies H, Stratton  MR,  Campbell PJ (2017). Universal patterns of selection in cancer and somatic tissues. Cell.

[18] Sui S, An X, Xu C, Li Z, Hua Y, Huang G, Sui S, Long Q, Sui Y, Xiong Y (2020). An immune cell infiltration-based immune score model predicts prognosis and chemotherapy effects in breast cancer. Theranostics.

[19] Mikami S, Mizuno R, Kondo T, Shinohara N, Nonomura N, Ozono S, Eto M, Tatsugami K, Takayama T, Matsuyama H (2019). Clinical significance of programmed death-1 and programmed death-ligand 1 expression in the tumor microenvironment of clear cell renal cell carcinoma. Cancer Sci.

[20] Picard E, Verschoor CP, Ma GW, Pawelec G (2020). Relationships between immune landscapes, genetic subtypes and responses to immunotherapy in colorectal cancer. Front Immunol.

[21] Goodman AM, Sokol ES, Frampton GM, Lippman SM, Kurzrock R (2019). Microsatellite-stable tumors with high mutational burden benefit from immunotherapy. Cancer Immunol Res.

[22] Li L, Goedegebuure SP, Gillanders WE (2017). Preclinical and clinical development of neoantigen vaccines. Ann Oncol.

[23] Schizas D, Charalampakis N, Kole C, Economopoulou P, Koustas E, Gkotsis E, Ziogas D, Psyrri A, Karamouzis MV (2020). Immunotherapy for pancreatic cancer: a 2020 update. Cancer Treat Rev.

[24] Kneip C, Schmidt B, Seegebarth A, Weickmann S, Fleischhacker M, Liebenberg V, Field JK, Dietrich D (2011). SHOX2 DNA methylation is a biomarker for the diagnosis of lung cancer in plasma. J Thorac Oncol.

[25] Schmidt B, Liebenberg V, Dietrich D, Schlegel T, Kneip C, Seegebarth A, Flemming N, Seemann S, Distler J, Lewin J (2010). SHOX2 DNA methylation is a biomarker for the diagnosis of lung cancer based on bronchial aspirates. BMC Cancer.

[26] Zhang YA, Zhou Y, Luo X, Song K, Ma X, Sathe A, Girard L, Xiao G, Gazdar AF (2016). SHOX2 is a potent independent biomarker to predict survival of WHO grade II-III diffuse gliomas. Ebiomedicine.

[27] Schneider KU, Dietrich D, Fleischhacker M, Leschber G, Merk J, Schaper F, Stapert HR, Vossenaar ER, Weickmann S, Liebenberg V (2011). Correlation of SHOX2 gene amplification and DNA methylation in lung cancer tumors. BMC Cancer.

