# Supplementary material for: A potential immunotherapeutic and prognostic biomarker for multiple tumors including glioma: SHOX2
Source: Hereditas. 2023 May 11;160:21. doi: 10.1186/s41065-023-00279-8 (PMC10173633; doi:10.1186/s41065-023-00279-8)
Supplement: Supplementary file 1 — Additional file 1: Supplementary Figure 1. Correlation between SHOX2 mRNA expression and prognosis of pan-cancers. Supplementary Figure 2. KEGG enrichment analyses of SHOX2-related genes in glioma. Supplementary Figure 3. Correlations between SHOX2 expression and ICP genes in glioma. Supplementary Figure 4. The correlations between SHOX2 expression and TIICs via ImmuCellAI in CGGA database. Supplementary Figure 5. Analysis showed the correlation between SHOX2 expression and ESTIMATE score in glioma. [file 41065_2023_279_MOESM1_ESM.docx]

**Supplementary materials**


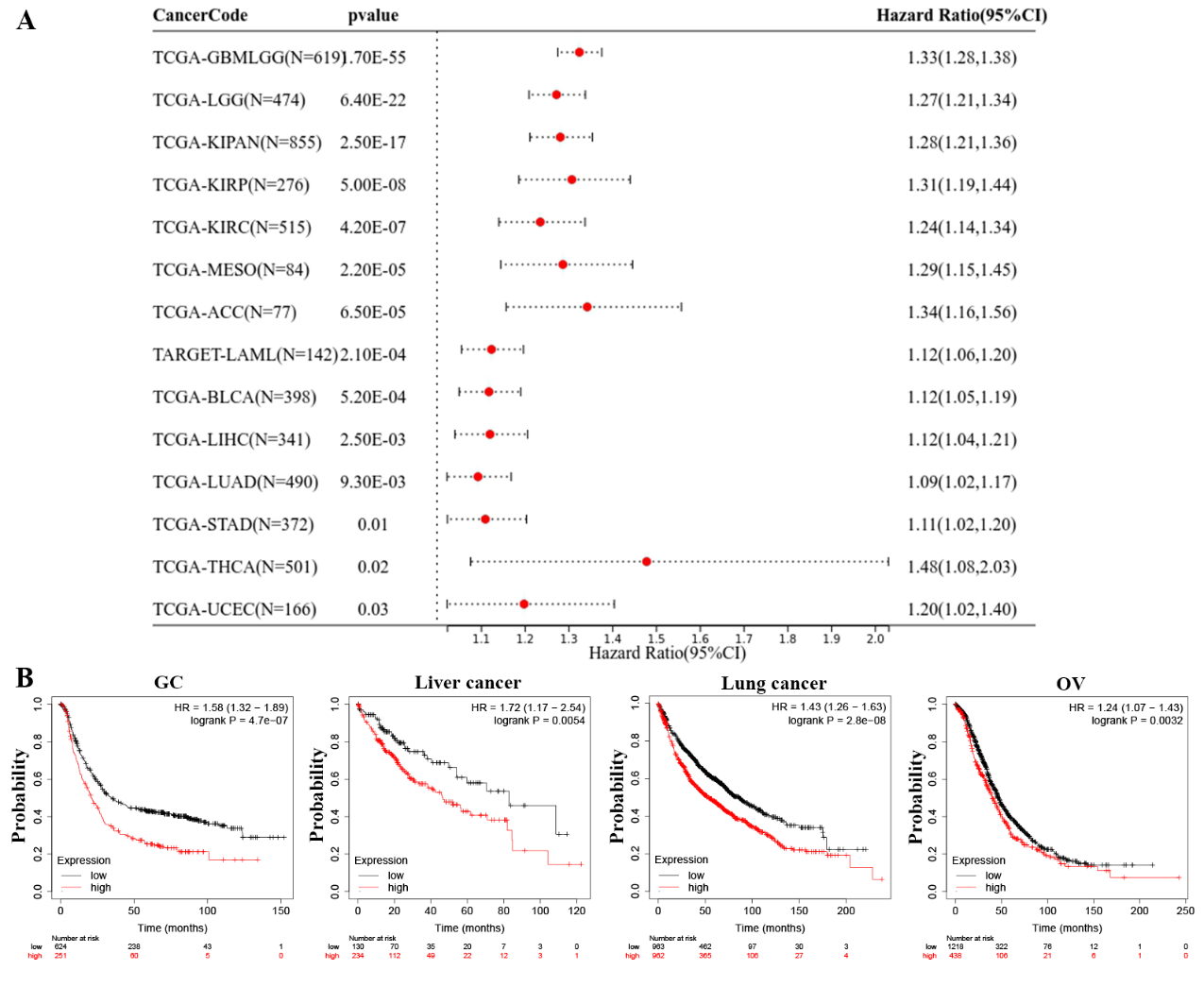


**Supplementary Figure 1. Correlation between SHOX2 mRNA expression and prognosis of pan-cancers. (A)** The relationships between SHOX2 expression and OS prognosis of multiple cancers via SangerBox. **(B)** The correlations between SHOX2 expression and OS prognosis of GC, liver cancer, lung cancer and OV via the Kaplan–Meier plotter.

**
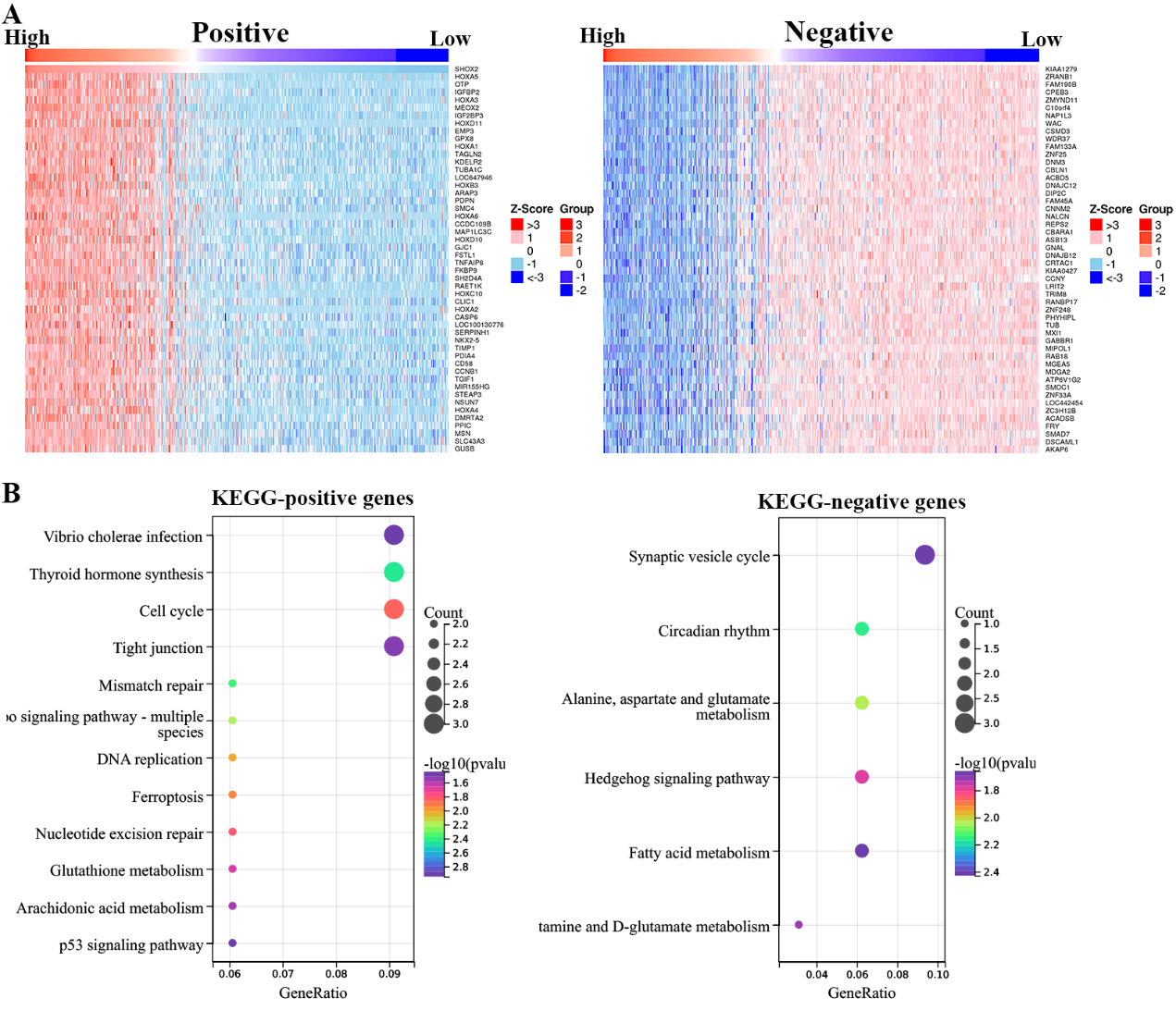
**

**Supplementary Figure 2. KEGG enrichment analyses of SHOX2-related genes in glioma. (A)** Heatmaps showed the top 50 positively-related genes or negatively-related genes of SHOX2 in glioma via LinkedOmics. **(B)** KEGG enrichment analyses of SHOX2 positively-related genes or negatively-related genes in glioma.

**
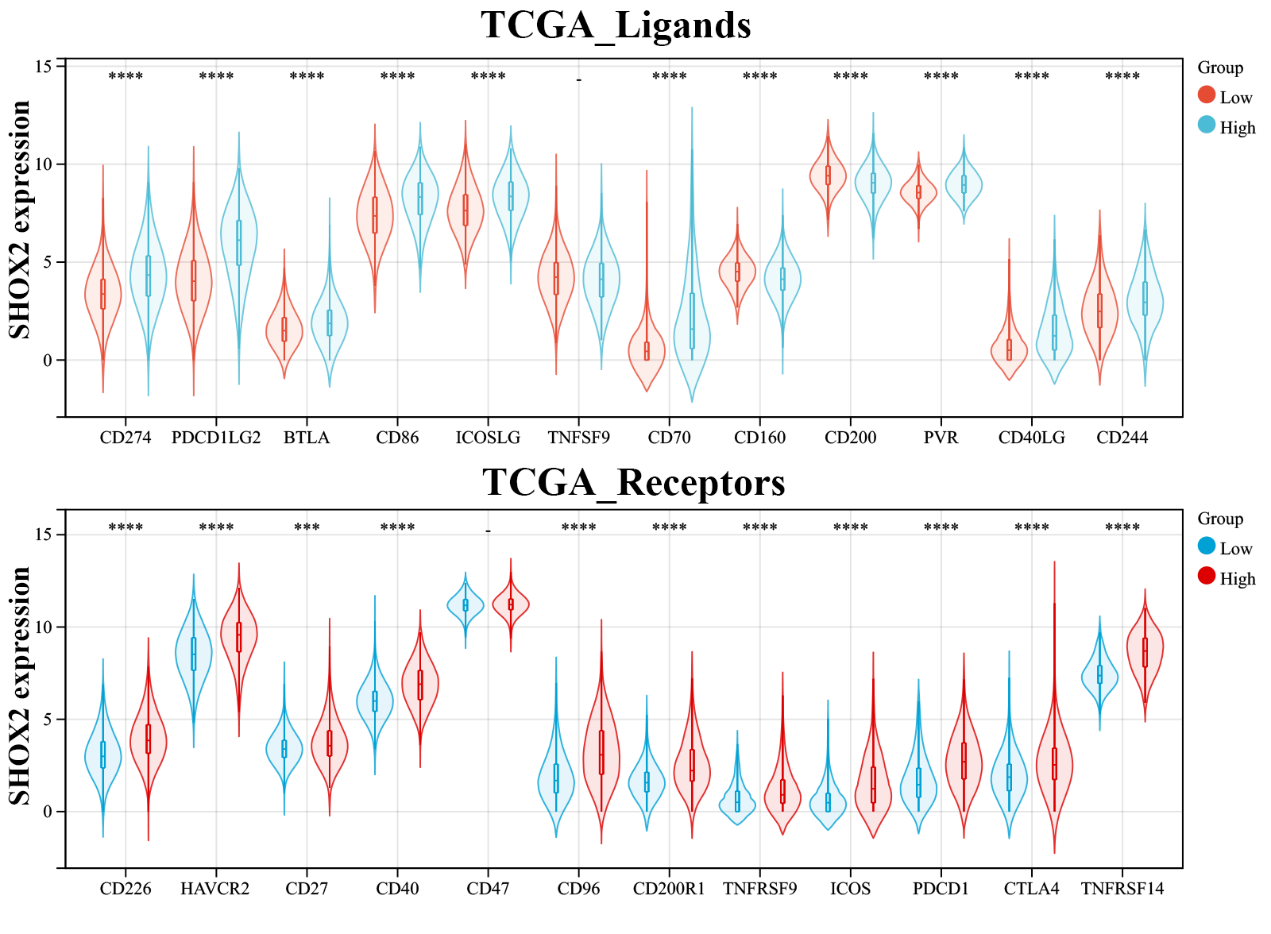
**

**Supplementary Figure 3. Correlations between SHOX2 expression and ICP genes in glioma.** The correlation between SHOX2 expression and ICP ligands and ICP receptors in glioma via TCGA database.


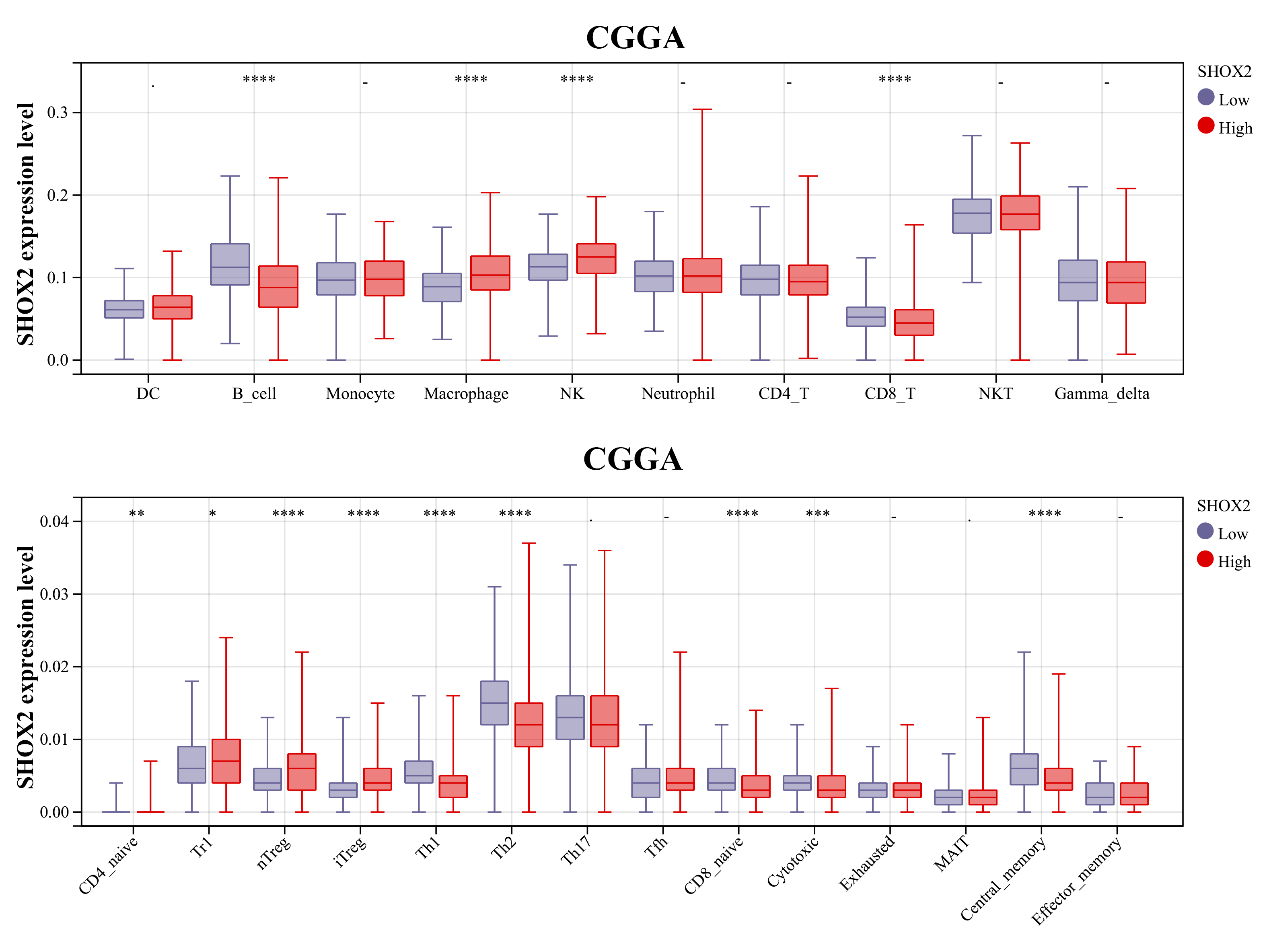


**Supplementary Figure 4. The correlations between SHOX2 expression and TIICs via ImmuCellAI in CGGA database.**

**
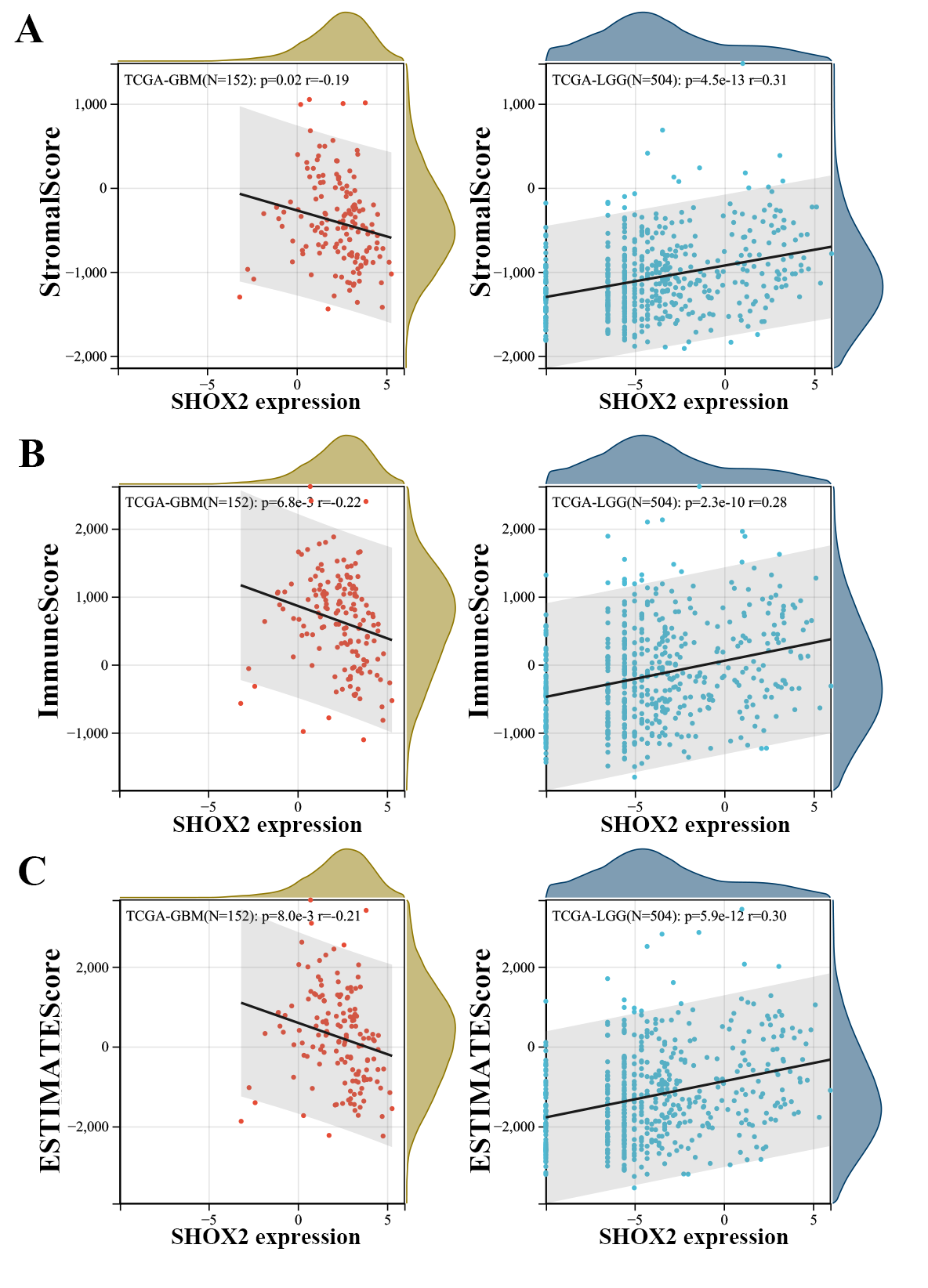
**

**Supplementary Figure 5. Analysis showed the correlation between SHOX2 expression and ESTIMATE score in glioma.** The correlation between SHOX2 expression and stromal score **(A),** immune score **(B)** and ESTIMATE score **(C)** in GBM and LGG via SangerBox.
